# An examination of teachers’ strategies to foster student engagement in blended learning in higher education

**DOI:** 10.1186/s41239-021-00260-3

**Published:** 2021-05-10

**Authors:** Géraldine Heilporn, Sawsen Lakhal, Marilou Bélisle

**Affiliations:** 1 Département de Pédagogie, Faculté d’Éducation, Université de Sherbrooke, 2500, boulevard de l’Université, Sherbrooke, QC J1K 2R1 Canada; 2 Centre de Recherche Interuniversitaire sur la Formation et la Profession Enseignante (CRIFPE-Sherbrooke), Sherbrooke, Canada

**Keywords:** Higher education, Student engagement, Blended learning, Teachers’ strategies

## Abstract

This qualitative study examined how teachers fostered student engagement in blended learning (BL), i.e., blended, blended online, and blended synchronous courses that combine synchronous and asynchronous activities. Twenty semi-structured interviews with teachers in various disciplines, at the undergraduate or graduate level in four universities, were conducted and analyzed using an inductive approach. Therefore, the study proposed a broad and comprehensive picture of teachers’ strategies to enhance student engagement in BL, that were classified in three meta-categories concerning (i) the course structure and pace; (ii) the selection of teaching and learning activities; and (iii) the teacher’s role and course relationships. Strategies were also linked with student engagement dimensions (behavioral, emotional, cognitive), whenever possible. The findings particularly emphasized the importance of a well-structured and -paced course, fully exploiting and integrating synchronous and asynchronous modes of BL. Clearly communicating how the course would unfold and corresponding expectations as well as establishing trusting relationships at the beginning of the semester also appeared as key to foster student engagement in BL. The use of various digital tools was also highlighted to promote student behavioral and emotional engagement at the undergraduate level, whereas cognitive and emotional engagement of graduate students was mainly targeted through experience-sharing and learning co-construction between students.

## Introduction

As a key component of student success in higher education, student engagement has received much attention in the last decade from administrators, practitioners, and researchers alike (Burke, 2019; Kahu & Nelson, 2018; Lee, 2014). Considered as "the holy grail of learning" by Sinatra et al. (2015, p. 1), it has indeed important repercussions on perseverance, in-depth learning, student satisfaction, and academic success (Christenson et al., 2012; Halverson & Graham, 2019; Kahu, 2013; Mandernach, 2015). Furthermore, student engagement is influenced by contextual variations such as learning environments or strategies deployed by teachers (Bond & Bedenlier, 2019; Fredricks et al., 2004, 2019; Kahu, 2013). As such, it is important to investigate how it can be fostered by teachers in specific learning environments.

Blended learning (BL) environments, which combine synchronous and asynchronous activities and are situated on a continuum between face-to-face and online teaching and learning, are of particular interest since the literature suggests that they have the potential to optimize student engagement (Graham, 2019; Halverson & Graham, 2019; Manwaring et al., 2017). Aiming to merge the benefits of synchronous interactions and online flexibility in terms of time, place, or even study pace, BL is also becoming increasingly popular in higher education thanks to the progress of digital technologies (Boelens et al., 2017, 2018; Johnson, 2019; Seaman et al., 2018). Despite suggestions of enhanced student engagement in BL, few studies in BL have specifically addressed student engagement in their research questions, and even less so teachers’ strategies to promote student engagement (Graham, 2019; Halverson et al., 2014; Siemens et al., 2015; Taylor et al., 2018). Nevertheless, teachers play a central role in BL, from course design to facilitation of interactions and support of students’ learning processes (Boelens et al., 2017; McGee & Reis, 2012). Therefore, there is a need to document how teachers foster student engagement in such environments. In order to fill this knowledge gap, the present study investigates teachers’ strategies to foster student engagement in BL in higher education.

## Conceptual framework, literature review, and research questions

### Student engagement

Recognized as a complex and multifaceted construct, student engagement is rooted in action (Bond et al., 2020; Kahu, 2013; Reschly & Christenson, 2012). Here considered in a course context, it represents the investment and energy that students devote to learning (Borup et al., 2020; Fredricks et al., 2016; Skinner & Pitzer, 2012). Often described as a multidimensional psycho-social process, numerous authors (Bond et al., 2020; Christenson et al., 2012; Fredricks et al., 2019; Kahu, 2013; Lawson & Lawson, 2013; Manwaring et al., 2017; Schindler et al., 2017) refer to the definition provided by Fredricks et al. (2004) based on a qualitative literature review. These authors define student engagement as having three interrelated dimensions: behavioral, emotional, and cognitive. In a course, *student behavioral engagement* concerns their participation in activities and compliance with rules or norms. Next, *student emotional engagement* refers to their emotional reactions to activities, peers, and the teacher, and their sense of belonging to the course. Finally, *student cognitive engagement* corresponds to their psychological investment in activities to master complex knowledge, as well as their use of learning or metacognitive strategies. Christenson et al. (2012) summarize student engagement by stating that “engaged students do more than attend or perform academically; they also put forth effort, persist, self-regulate their behavior toward goals, challenge themselves to exceed, and enjoy challenges and learning” (p. v).

### Blended learning

BL is situated on a continuum between face-to-face and online learning (Lakhal & Meyer, 2019). The Handbook of Blended Learning defined BL as a combination of face-to-face and online activities (Bonk & Graham, 2012), while some more precise definitions explicitly identify a decrease in face-to-face meetings (Bates, 2018; McGee & Reis, 2012; Picciano, 2009), e.g., between 30 and 79% of online learning (Allen & Seaman, 2016). Although this point is not necessarily made explicit in the literature, the present study considers that such a decrease should be inherent to BL so as to avoid a one-and-a-half course phenomenon (McGee & Reis, 2012).

Given the aim of BL to combine the benefits of synchronous interactions with online flexibility and considering the improvements in digital technologies, new BL environments that allow synchronous activities to happen online instead of face-to-face for all or part of the students have also emerged in the last 15 years (Lakhal et al., 2017, 2020). The literature describes three common BL environments: ‘Traditional’ Blended, Blended Online, and Blended Synchronous courses (Lakhal & Bélisle, 2020; Lakhal et al., 2020; McGee & Reis, 2012). First, *Traditional Blended Courses* combine face-to-face with asynchronous online T&L activities. Second, *Blended Online Courses* combine synchronous and asynchronous online T&L activities (Power, 2008; Power & Vaughan, 2010). Also found in the online learning literature, they are part of BL since synchronous online meetings enable real-time interactions between students and teachers, as is the case for face-to-face meetings. Finally, *Blended Synchronous Courses* combine asynchronous online with synchronous face-to-face/online activities where on-campus/remote students simultaneously participate (Bower et al., 2015; Lakhal et al., 2017, 2020; Raes et al., 2019, 2020). In some cases, students may also have the possibility to watch meeting recordings or alternative videos instead of participating in synchronous T&L activities, thus being offered full flexibility of participation corresponding to HyFlex Courses (Beatty, 2007, 2014, 2019).

### Student engagement in blended learning

Through the combination of asynchronous and synchronous modes, BL environments can bring together various teaching and learning activities while facilitating differentiated and personalized instruction (Boelens et al., 2018; Taylor et al., 2018). Merging the benefits of synchronous and asynchronous communication, BL aims at “extending thinking and discourse over time and space” and is “specifically directed to enhancing [student] engagement” (Vaughan et al., 2013, p. 9) while leveraging digital technology opportunities in a learner-centered approach (Garrison & Vaughan, 2008; Taylor et al., 2018). Therefore, numerous authors identify BL as a fertile ground to optimize student engagement (Halverson & Graham, 2019; Halverson et al., 2014; Manwaring et al., 2017; Serrano et al., 2019; Spring et al., 2016; Taylor et al., 2018).

In a literature review, Halverson et al. (2014) mentioned that the term ‘engagement’ was often used in BL publications although it was rarely defined. Nevertheless, these authors and other recent literature reviews indicated that there were few studies focusing specifically on student engagement in BL (Halverson et al., 2014; Martin et al., 2017; Pima et al., 2018; Raes et al., 2019). Most existing publications also investigated single courses (e.g., Binnewies & Wang, 2019; Cundell & Sheepy, 2018; Raes et al., 2020), specific activities (e.g., Foogooa & Ferdinand-James, 2017; Kulkarni & Iwinski, 2016; Tan & Hew, 2016), or aspects of student engagement (e.g., Berry, 2019; Truhlar et al., 2018). Consequently, given the potential for enhanced student engagement in BL, numerous authors have called for pursuing research on student engagement in BL (Drysdale et al., 2013; Graham, 2019; Halverson et al., 2014; Henrie et al., 2015; Manwaring et al., 2017).

### Teachers’ strategies to foster student engagement in blended learning

Student engagement being malleable through pedagogy, it can be influenced by teachers’ strategies, i.e., what teachers do to encourage student engagement in their courses (Fredricks et al., 2004, 2019; Kahu, 2013; Lawson & Lawson, 2013). In BL environments, there are however few studies investigating what teachers do and why they do it (Smith & Hill, 2019; Taylor et al., 2019; Torrisi-Steele & Drew, 2013), and even fewer investigating how they foster student engagement (Halverson & Graham, 2019; Halverson et al., 2014; Jeffrey et al., 2014; Manwaring et al., 2017; Siemens et al., 2015; Taylor et al., 2018). For instance, although the literature stipulates that synchronous and asynchronous modes of BL must be thoughtfully integrated in order to optimize student engagement (e.g., Garrison & Vaughan, 2008; McGee & Reis, 2012), several authors mentioned that there are few concrete recommendations in this regard (Graham et al., 2014; Manwaring et al., 2017; Siemens et al., 2015; Taylor et al., 2018). The sparse literature concerning how teachers foster student engagement in BL addresses the issue with varying degrees of specificity, ranging from specific digital technology applications to activities to general strategies.

The following literature review focuses on teachers’ perspectives relating to strategies fostering student engagement in BL. Excluding course case studies, Vaughan (2014) studied the role of online collaborative learning applications to foster student engagement and success in traditional BL courses for 1st-year undergraduates. By way of a mixed methodology notably involving teachers’ interviews (n = 8), the author concluded that such applications enhanced student engagement. However, he suggested that future research should explore whether the use of digital technologies stimulates student engagement in BL in and of itself or instead mediates a more general strategy such as active and collaborative learning. Montgomery et al. (2015) also examined the role of digital technologies in fostering student engagement in three traditional BL courses for education undergraduates, through teachers’ narratives (n = 3). The teachers reported that students were first engaged asynchronously online using varied resources (e.g., texts, videos), choices being provided to foster student engagement. In subsequent synchronous meetings, student engagement was sustained through active learning, sometimes using an experiential approach. Then student engagement was reinforced online by individual or collaborative projects and digital resources devoted to deepening understanding of the contents. Finally, the authors stressed the importance of student-content interactions to promote student engagement. Although this publication described several teachers’ strategies fostering student engagement in BL, a detailed analysis of strategies shared by the three courses was not provided and the number of courses was limited, which could be interpreted as methodological limitations.

In a larger study, Jeffrey et al. (2014) examined how teachers in traditional BL courses for business undergraduates fostered student engagement, both face-to-face and asynchronously online (n = 9). The findings revealed that student engagement was encouraged more in face-to-face meetings than online, with few teachers having developed asynchronous online activities (e.g., quizzes) to promote student engagement. Three teachers also monitored student engagement online through assignment submission, enabling them to "re-engage" some students through personalized emails. Despite these initiatives, the teachers’ strategies to foster student engagement were much more developed in face-to-face than online mode. The authors concluded that enhancing student engagement requires that teachers fully exploit the potential of BL by integrating various asynchronous and synchronous activities. More recently, Heilporn and Lakhal (2020) investigated teachers’ strategies (n = 8) to foster graduate students’ engagement in traditional BL courses in business. Using semi-structured interviews and content analysis of course platforms, the authors first noted that most teachers emphasized emotional, behavioural, or cognitive engagement of students depending on the perception of their role in the course. Next, the findings showed that some teachers had divided their course content between synchronous and asynchronous modes without any actual reflection or integration between activities. In contrast, other teachers designed asynchronous activities based on the transmission of content knowledge that was later integrated in synchronous meetings. According to the teachers, such a strategy fostered student engagement. Regardless of the previous findings, most teachers promoted student engagement asynchronously online through quizzes, discussion forums, videos, and various news articles. Finally, during synchronous meetings, teachers indicated that they fostered student engagement through active and collaborative activities related to the business practice. Where appropriate, synchronous activities following an experiential approach (prepared asynchronously online) also stimulated student engagement in BL courses.

## Research questions

There are few studies specifically addressing student engagement in BL, and even fewer concerning teachers’ strategies to foster student engagement in such environments. While studies presented in the previous section have paved the way for research about this subject, the examination of teachers’ strategies in these studies was limited by the number of teachers included and their specific focus (e.g., on digital technology applications) or context (e.g., business faculty). Teachers’ strategies were also studied with varying degrees of specificity, which emphasizes the need to classify these in a clear and organized way. Moreover, most publications did not present an explicit definition of student engagement and only Heilporn and Lakhal (2020) investigated the issue using a multidimensional perspective. Furthermore, almost all studies concerned undergraduate courses, and all were situated in traditional BL courses. In blended online or blended synchronous courses, in particular, the question of how to optimize student engagement is still open (Raes et al., 2020). As a result, the way in which teachers foster student engagement in BL environments (traditional blended, blended online, or blended synchronous) has yet to be studied (Graham, 2019; Raes et al., 2020; Siemens et al., 2015). Hence, the following general research question was addressed in this study: What strategies do teachers use to foster student behavioral, emotional and cognitive engagement in BL?

## Method

### Participants

In order to develop a broad picture of strategies that teachers use to foster student engagement in BL, the research study was conducted in various disciplines and in several universities across the province of Quebec (Canada), diversifying sources to enhance the external validity of our findings (Merriam & Tisdell, 2015). Four universities offering BL courses were contacted during fall 2019 and were included in this study.

The selection of participants was purposeful and targeted teachers (either professors or lecturers) that were identified through referrals from instructional designers as having at least two semesters of experience in BL and showing an interest in pedagogy. Twenty teachers participated in the study, with an equal number of men and women. At the time of the interview, they had between two and twenty-two years of teaching experience in higher education and from two to fifty semester experiences in BL. They taught in various disciplines, from social sciences and humanities (e.g., education, sociology, marketing, communication) to natural sciences and engineering (e.g., mathematics, computer science, economy, accounting). Teachers were coded P1 to P20.

### Data collection

Ethical approval was granted by the ethics board committees at the authors’ university and at other universities included in the study. Prior to data collection, an interview guide was developed in order to lead the conversation while allowing flexibility in participants’ responses (Merriam & Tisdell, 2015). The clarity and relevancy of each question were evaluated by four experienced researchers, which helped us to improve the interview guide. A pretest of the interview was then conducted with a teacher in a blended course who happened to be an experienced qualitative researcher. Thereafter, all participants were sent the main questions and a brief definition of behavioral, emotional, and cognitive engagement prior to their interviews. Semi-structured individual interviews were conducted during early winter 2020. All participants signed a written consent for their participation and agreed to an audio or video recording of their interview. Each interview lasted about an hour, as previously agreed by participants. Following general questions regarding their teaching experience, the teachers were asked to focus on a BL course they had taught. As shown in Table 1, course characteristics in terms of education level, BL environment and number of students varied greatly between the interviewees (see detailed information in Appendix A).

**Table 1:** BL course characteristics (n = 20)

	Undergraduate	Graduate
Education level	13	7
BL environment	5 TBL, 2 BOL, 6 BSL	5 TBL, 2 BOL
Number of students	45–130 TBL, 25–100 BOL, 50–300 BSL	20–50 TBL, 7–15 BOL

*TBL* traditional blended learning, *BOL* blended online learning, *BSL* blended synchronous learning

During the interview, participants were first asked to explain how their course was organized, a question aimed at gathering preliminary information about the sequencing of synchronous and asynchronous activities. Next, the interviewer briefly explained the three dimensions of student engagement (behavioral, cognitive and emotional) and asked the participants to link, whenever possible, their following answers to these dimensions. Then, they were asked to talk about course situations where they noticed high student engagement concerning any of the three dimensions and how they fostered or maintained it. They were also asked to talk about situations where they noticed low student engagement regarding any of the three dimensions and how they managed to re-engage the students. As also a teacher in blended courses, the interviewer ensured to keep an objective and neutral stance, for instance using very opened questions to ask participants for more details (Merriam & Tisdell, 2015). The questions were also formulated as simply as possible, avoiding any specific educational or pedagogical vocabulary, given the diversity of disciplinary specialties of participants (Merriam & Tisdell, 2015). Moreover, the fact that most participants were not researchers in education nor familiar with the dimensions of student engagement sometimes hindered to gather information about which dimensions they targeted in some situations. While asking for clarifications to enrich participants’ explanations, the interviewer stayed neutral to avoid introducing bias in the data and kept in mind that they could have no answer (Merriam & Tisdell, 2015).

### Data analysis

Following their full transcription, the interviews were analyzed through a general inductive approach (Miles et al., 2020; Thomas, 2006) and coded using Nvivo 11. Specifically, Thomas (2006) described the following coding steps: initial reading of transcripts, identification of data segments related to objectives, labelling segments into emerging categories, refining categories to avoid redundancies, highlighting the main categories. First, close and iterative reading of transcripts enabled to identify meaningful data segments aimed at answering the general research question. Initial codes corresponding to teachers’ strategies were generated from data listening and reading as well as the interviewer’s notes, while taking existing strategies from the literature into account. Then data segments were assigned codes in the Nvivo program using concept coding (Miles et al., 2020). Throughout the coding process, codes were revised, refined, and hierarchized to minimize redundancies and overlaps (Thomas, 2006). Each code was labelled and assigned an operational definition that was clarified throughout the coding and analysis process (Miles et al., 2020). To assess the trustworthiness of the coding frame, a second and independent researcher was given a description of each code and a sample of raw data segments, then asked to assign the data segments to codes. The interrater reliability was 90% (45/50). The second researcher next coded some transcripts, then asked the first researcher to assign raw data segments to codes, which yielded the same interrater reliability and confirmed a high level of inter-coder agreement. Researchers also discussed the coding frame to ensure a conceptual and structural unity (Miles et al., 2020). Following the first cycle of data condensation and discussion between researchers, codes were further refined and clustered into categories of teachers’ strategies (Miles et al., 2020). The final coding scheme is described in Appendix B. For instance, the teachers’ strategies that consisted in (a) providing clear indications about when, what and where activities were to be completed, (b) making explicit connections between synchronous and asynchronous modes and (c) ensuring a continuity between activities throughout the semester were grouped into a first category of strategies related to the course structure. Whenever possible (depending on the level of detail of the explanations provided by the participants), categories of strategies and specific teachers’ strategies were linked to student behavioral, emotional, or cognitive engagement according to Fredricks et al.’s (2004) definition of the dimensions and their operational indicators as provided by Bond et al. (2020). In-depth data analysis and writing were performed through a recursive comparison process between data (transcripts and interviewer’s notes), coding, and interpretation, enabling the capture of recurring patterns of data (Merriam & Tisdell, 2015). The diversity of sources (participants, disciplines and course characteristics) also allowed us to obtain a broad and comprehensive picture of teachers’ strategies.

## Results

Following in-depth data analysis, teachers’ strategies to foster student engagement in BL environments were classified into three meta-categories concerning (i) the course structure and pace; (ii) the selection of teaching and learning activities; and (iii) the teacher’s role and course relationships. These are detailed in the next sections.

### Course structure and pace

**Fig. 1:**
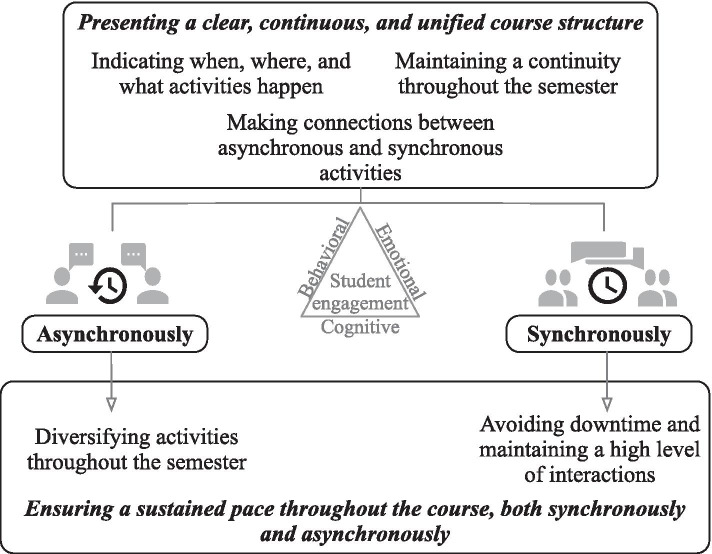
Teachers’ strategies relative to course structure and pace

Since BL implies that the students navigate between synchronous (face-to-face or online) and asynchronous modes, most teachers emphasized that course structure and pace were key to fostering student engagement. Two categories of strategies are illustrated in Fig. 1 and hereafter explained.

First, most teachers fostered student behavioral and emotional engagement by *presenting a clear, continuous, and unified course structure*. This involved indicating when, what, and where (face-to-face, synchronously online, or asynchronously online) activities were to be completed. “Behaviorally, I've structured the course so that it's very easy to follow and the students don't get lost or waste time. […] Giving them a structure, keeping it the same throughout the semester, I find it reassures them” (P1).

Most teachers also fostered student engagement by making connections between asynchronous and synchronous activities, explicitly emphasizing their interrelations. They planned synchronous meetings (face-to-face or online) to complement asynchronous activities. This way, the students perceived the added value of attending synchronously, which, in turn, generated positive emotional reactions, thereby fostering behavioral and emotional engagement. Synchronous meetings were often structured so that students could actively integrate the content that was earlier addressed asynchronously, which helped them to deepen the course content and stimulated their cognitive engagement. “The idea is to review the most important content items, to highlight them; I think that helps students. They tell me it’s useful. […] It also allows me to provide a more nuanced instructional content” (P10). Some teachers also imposed specific preparations before meeting synchronously to increase students’ interest during those meetings, thus generating emotional engagement. After a synchronous meeting, some teachers further encouraged cognitive engagement through asynchronous assignments so that the students would continue deepening their understanding of the content, again recalling the interrelations between synchronous and asynchronous activities. Finally, several teachers explained that maintaining continuity between activities throughout the semester promoted student engagement. For instance, one teacher used concrete examples from one of her research projects as a guiding thread in asynchronous activities throughout the semester. She explained that students liked this approach and it helped to enhance their emotional as well as their cognitive engagement. Most teachers also proposed similar activities from one synchronous meeting to another, a strategy that encouraged student behavioral and emotional engagement since students knew what to expect.

*Ensuring a sustained pace throughout the course, both synchronously and asynchronously,* also stimulated student engagement. In asynchronous mode, teachers helped students to maintain a sustained pace in learning by diversifying activities throughout the semester, which fostered their behavioral and emotional engagement. “The key is diversity in activities of the modules. Ensuring it's always a little bit different. I feel like I'm going to pick different students and not exhaust them” (P9). Teachers explained that this helped students to go through the content, while balancing more and less demanding activities during a semester so as to take students’ individual preferences into account. Teachers also promoted student engagement by carefully planning synchronous (face-to-face or online) meetings to avoid downtime and maintain a high level of interaction. This enhanced student participation and yielded positive emotional reactions, hence their behavioral and emotional engagement. “The students are engaged from the moment they walk into class until the moment they leave. When they come out, they're exhausted. It's very demanding for them, but their mood is good” (P1).

Most synchronous online meetings (in BOL or BSL) were also at most 2 h long, compared to typical 3-h meetings in face-to-face mode. According to several teachers, meetings of this length kept students from becoming bored and helped to maintain their emotional and behavioral engagement.

The students appreciate the more concise format, so there are fewer exercises than I could assign in face-to-face meetings […] For instance, a case study would take less time than a case we'd do face-to-face; we get right to the point. Specific questions will be asked, an application, but shorter. (P2)

Most teachers also emphasized the need to be very dynamic and entertaining during synchronous online meetings so as to enhance student emotional engagement.

### Selection of teaching and learning activities

**Fig. 2:**
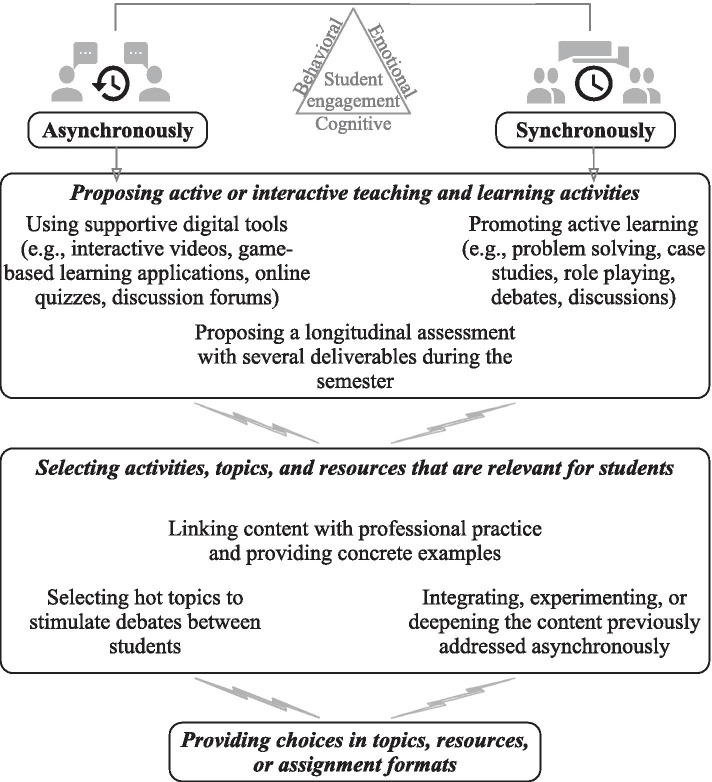
Teachers’ strategies relative to the selection of teaching and learning activities

Teachers also fostered student engagement by thoughtfully selecting activities, whether in asynchronous or synchronous mode. Three categories of strategies are illustrated in Fig. 2 and detailed below.

Teachers promoted student engagement by *proposing active or interactive teaching and learning activities.* In asynchronous mode, most teachers indicated using supportive digital tools (e.g., commented slides, interactive videos, game-based learning applications, online quizzes, discussion forums) to stimulate student participation and attention, therefore student behavioral engagement, particularly at the undergraduate level. “I designed an interactive capsule. It's something that came out, that they liked. They told me that the interactive aspect helped them get through the whole capsule, and it's something they appreciate, so that's what got their engagement” (P15). In undergraduate courses, most teachers also used frequent online assessments (e.g., contributions in discussion forums, quizzes) to ensure that the students stayed active asynchronously, thus stimulating their behavioral engagement. One teacher noted that there could be more innovative digital tools, but she did not see their relevance or feasibility. Furthermore, most teachers also fostered student engagement by proposing a longitudinal assessment with several deliverables during the semester, thus enabling students to apply and deepen their understanding of the content while improving themselves incrementally. The summative deliverables forced student participation while the longitudinal aspect and the teachers’ recurrent feedback stimulated their psychological investment, thereby promoting student behavioral and cognitive engagement.

In synchronous meetings, most teachers emphasized that promoting active learning enhanced student behavioral engagement. Depending on the course content, these were problem-solving activities, case studies, role-playing sessions, or discussions in teams or with the whole group. When appropriate, teachers enhanced student engagement by prompting debates between students. For instance, students would discuss a topic or study a case in teams and then share their conclusions with the whole group. While team discussions enhanced student behavioral engagement through participation, different conclusions then generated whole group debates and promoted student cognitive engagement. At the undergraduate level, several teachers also enhanced student participation and attention during synchronous meetings by using poll interactive applications (e.g., Kahoot, Socrative), again fostering the students’ behavioral engagement.

Pretty much everyone participates, and I’ve noticed that they’re really more attentive when I use the questions on Socrative, versus the same questions on PowerPoint with answers by a show of hand. I’ve seen a really big difference in behavior. So that helped; it’s a remarkably simple digital tool, but the students really liked it. (P8)

Some teachers also proposed problem-solving type assessments during or following a synchronous meeting. Such a strategy promoted both student participation in previous asynchronous activities and knowledge deepening in synchronous meetings, which encouraged their ongoing behavioral and cognitive engagement.

*Selecting activities, topics, and resources that are relevant for students* also stimulated their engagement in the course. Teachers indicated that linking content with professional practice fostered student emotional and cognitive engagement, several insisting on the importance of also providing concrete examples in asynchronous activities. A majority of teachers also explained that experts talking about their professional practice stimulated student emotional engagement. Even though these people could be invited for synchronous meetings, some teachers created experts’ videos when teaching a course for several semesters. One teacher also fostered student cognitive engagement by combining an expert’s videos and a synchronous discussion with that person. Furthermore, several teachers enhanced student engagement by selecting hot topics to stimulate debates, whether synchronously or asynchronously.

Often, debates can be prompted by the news, and I really feel that there is a fairly strong engagement both from the online students who start writing their thoughts quickly and from the people who are in face-to-face. At that point, I must make sure that everyone is able to speak regardless of whether they are physically present or at a distance. I think that this makes the class more dynamic. (P2)

One teacher also indicated that he used different topics to better engage the diverse student population in his course, explaining that topics or questions that unsettled the students also stimulated their engagement. Finally, most teachers enhanced the relevance of synchronous activities with a focus on integrating, experiencing, or deepening the content previously addressed asynchronously. Applying or experimenting with elements of the course content stimulated student behavioral engagement through participation, while integrating and deepening the content promoted their cognitive engagement.

They discover the content before we meet, and then we go further, either going deeper or experimenting. If we experiment, they’re necessarily engaged because they're in action. If we go deeper, most of the time I’ll try to put the content into practice or apply it through discussions. So their engagement comes through experience-sharing, or co-learning. (P15)

In graduate BL courses, several teachers invited students to find their own individual learning goal, other than the course objectives, to enhance emotional and cognitive engagement from the beginning of the semester.

I let them know that the course is for them; they aren’t doing it for me. This is a way to entice them, emotionally speaking. Then, I try to engage them cognitively by highlighting the course challenges that are linked to their future profession, to explain that they’ll have to think according to specific authentic situations. I think that this approach interests them and makes them want to put in the effort; they’ll want to do the activities. (P20)

Graduate BL courses mostly relied on experience-sharing to stimulate student emotional and cognitive engagement. While the students co-constructed their learning about professional practice, the teacher provided an external view with respect to the topics discussed and ensured students’ reflection gradation, thus fostering their cognitive engagement.

Finally, *providing choices in topics, resources, or assignment formats* also fostered student engagement, whenever appropriate. Several teachers indicated that allowing students to select their preferred topic for a discussion or an assignment, either freely or from a predefined list, enhanced their emotional and cognitive engagement.

I offered them a fairly wide selection of subject matter to address during the class, a list of 15 issues, and I kept the 3 most popular ones […] They liked this approach because they felt being in control of the class. They were all well prepared, participated appropriately, and had synthesized the topic well. (P9)

Some teachers also spoke of providing choices about the format in which to submit an assignment (e.g., text-based or video) to foster student emotional engagement. At the graduate level, one teacher also explained that teams of students had to animate discussion forums for 1 week during the semester.

They have to choose a case, either encountered in real life or in their work, or they build upon several experiences of the team members. They post their case on the forum and ask 3 or 4 questions to the group, then for 15 days they’ll be in charge of directing this forum. They have to respond to each intervention, relaunch the debate […] So they're responsible for facilitating. Of course, during the time they act as facilitators, they’re engaged as interested parties, but it’s also in their interest to participate in other teams’ debates because otherwise they run the risk that others will not participate when they’re facilitating. (P15)

Positioning the students in charge of the content in a leading position, while acting as facilitators whenever needed, enhanced student cognitive engagement.

### Teacher’s role and course relationships

**Fig. 3:**
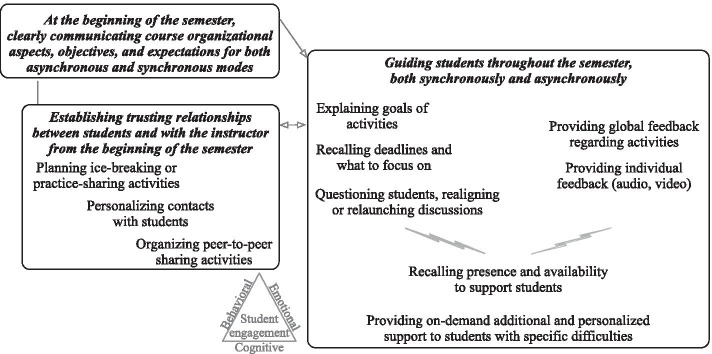
Teachers’ strategies relative to course relationships and their role

All teachers emphasized that guiding and supporting the students from the beginning and throughout the semester, whether in a large group, in teams, or individually, fostered student engagement. Three categories of strategies are illustrated in Fig. 3 and explained below.

*At the beginning of the semester*, *clearly communicating course organizational aspects, objectives, and expectations for both asynchronous and synchronous modes* fostered student behavioral and emotional engagement in BL.

Before the course starts, I send a welcoming email to the students that includes a course plan and an explanation of how everything works. Really, step by step, and I repeat the procedure during the first face-to-face class. So it’s really to explain in great detail how we have to work. (P19)

Explicitly telling students how the BL course was organized, with both asynchronous and synchronous activities, encouraged their behavioral and emotional engagement. In undergraduate BL courses, in particular, several teachers indicated that making the students feel secure fostered their emotional and behavioral engagement, which is why some of them spent one or two synchronous meetings clearly explaining the course structure, organization, and communicate expectations. Clear communications at the beginning of the semester reduced student anxiety and negative emotional reactions while fostering their participation in activities. “I put a lot of attention into explaining exactly how the activity was going to happen, precisely because sometimes it's very clear to the teacher while the students are missing important information, which can make them exceedingly anxious” (P16).

Teachers also fostered student emotional engagement by *establishing trusting relationships between students and with the teacher from the beginning of the semester*. “It's a bit like a trust contract in that they perceive the person whom they're dealing with as the group leader and they're confident that everything is going to go well […] they're reassured that we'll communicate, and they’ll be able to ask questions” (P15). Several teachers used ice-breaking activities in the first synchronous meeting to develop a sense of belonging to the course, thus fostering student emotional engagement. Some of them invited the students to share course-related examples from their own practice, if applicable, in discussion forums, and reused these examples in subsequent synchronous meetings to demonstrate their trust in student experiences. Throughout the semester, personalized contacts between students and the teacher also nurtured their engagement in the course. Some instructors also enhanced students’ sense of belonging to a group and psychological investment by asking them to comment on another student’s work, thus fostering their emotional and cognitive engagement. “Having to comment on a peer adds to their engagement […] they realize that they’re not alone; they’re part of a class. And the fact that another student is waiting for their feedback also creates an additional commitment” (P9).

Finally, *guiding students throughout the semester, both synchronously and asynchronously,* promoted their engagement in the course. This involved explaining the goals of activities both asynchronously and synchronously in order to stimulate student participation and thus their behavioral engagement, especially at the undergraduate level.

For each course module, I clarify objectives and skills and specifically demonstrate how we’re going to achieve them. With a short video: "We're getting into that content; it's going to be useful for you for this reason." So I come to convince them or hook them a bit, hoping they'll participate. (P9)

Providing deadline and important topics reminders through asynchronous communication (e.g., emails or posts on the course platform) also fostered student behavioral and emotional engagement. This helped students to complete important activities while highlighting the teacher’s caring and concern, which they appreciated. Several teachers talked about reducing distance as much as possible, for instance by customizing audio and video resources. Some also provided additional short news flashes to pique students’ interest, thus stimulating their emotional engagement.

We send weekly emails to the students, for instance saying, "Hey don't forget to do that," and I also give them some additional news like "You heard that on TV; well, it's directly related to what we discussed." Very simple, short things. Students told me they liked this approach. They found it very interesting and engaging. (P9)

Teachers also encouraged cognitive engagement by questioning students, thus realigning or relaunching discussions both asynchronously (e.g., in discussion forums) and synchronously. More than merely answering questions, they tried broadening students’ discussions or reflections to promote their cognitive engagement. “I'm going to relaunch them by saying ‘Imagine, if this situation happens, we have to do this, and then what?’ I'm going to challenge them” (P17). Several teachers also mentioned that their own engagement in a course stimulated student behavioral and cognitive engagement, because it made students want to put in an equal amount of effort. Wherever appropriate, providing general feedback regarding a specific activity or assessment, either asynchronously or synchronously, fostered student cognitive engagement. Several teachers mentioned that giving positive feedback to the whole group also promoted student emotional engagement. Regarding individual feedback, some teachers fostered student emotional engagement by providing audio or video feedback, thereby enhancing their presence. “In terms of student engagement, the importance of feedback […] [is] that they understand the how and why of their results, while being congratulated for what they did well” (P17). Furthermore, providing additional and personalized on-demand support for students with specific difficulties promoted their emotional engagement. Teachers regularly reminded students of their availability to provide support, whether synchronously online or face-to-face, which helped students get through the semester.

There may be students with serious difficulties. I’d see them several times during the semester if necessary […] I tell them "Prepare your questions, I'll see you as many times as it takes." It can be by Skype, by email, depending on the nature of the problem. Those who come are super happy. (P15)

Whether online synchronously, face-to-face, or sometimes online asynchronously (by email), students were happy to get help. They felt supported and this reassured them.

## Discussion

In contrast to publications with a specific focus like collaborative learning applications (e.g., Vaughan, 2014) or a limited number of participants (e.g., Jeffrey et al., 2014; Montgomery et al., 2015), this study aimed to present a broad and comprehensive picture of strategies used by teachers to enhance student engagement in BL in higher education. While previous studies were mainly conducted at the undergraduate level (e.g., Vaughan, 2014), in specific disciplines (e.g., Jeffrey et al., 2014) and within traditional blended courses, this study covers a large array of disciplines and different BL environments at the undergraduate and graduate levels in four higher education institutions. Strategies used by teachers to foster student engagement in BL were identified for asynchronous and synchronous modes and, whenever possible, linked to student engagement dimensions (behavioral, emotional, cognitive). They were clearly organized in eight categories and three meta-categories ranging from a more external perspective (course structure and pace) to a more internal one (teacher’s role and course relationships), which are discussed below.

### Course structure and pace

From an external perspective, ensuring that BL courses are well-structured and -paced appeared as key to enhance student engagement. In line with McGee and Reis (2012), a clear course structure fostered student behavioral and emotional engagement. Regarding the thoughtful integration of synchronous and asynchronous activities recommended in BL (Garrison & Vaughan, 2008; Jeffrey et al., 2014; McGee & Reis, 2012), this study identified concrete strategies that teachers use to enhance student engagement, such as unifying the course structure by designing synchronous meetings (face-to-face or online) so that they complement asynchronous activities, making explicit connections between them, and maintaining a continuity throughout the semester. In this regard, some teachers having less experience in BL expressed a sense of uneasiness regarding student engagement in asynchronous online mode, while their courses demonstrated a lack of connections and continuity between asynchronous and synchronous modes. Since more experienced teachers generally made full use of both modes, it suggested that teachers need time to adjust and fully exploit the potential of BL. As Bruggeman et al. (2021) recommended, teachers have to fully understand what is BL, as well as taking some time to experiment and reflect on their courses while benefitting from professional support.

This study also highlighted that maintaining a sustained pace in both synchronous and asynchronous modes of BL fostered student behavioral and emotional engagement. Concrete strategies in this regard consisted in avoiding downtime and maintaining a high level of interaction in synchronous meetings, as well as diversifying activities throughout the semester in the asynchronous BL mode. Some specificities were also stressed regarding the pace of synchronous online meetings, usually shorter than face-to-face meetings and in which teachers had to focus on being very dynamic to promote student emotional engagement. To our knowledge, this is the first time that ensuring a sustained pace was explicitly emphasized to foster student engagement in the BL literature.

This first meta-category suggested that teachers take a step back to reflect on their BL course and ask themselves if it is adequately structured and paced, hence taking full advantage of the potential of BL and designing the course in such a way as to optimize student engagement (Garrison & Vaughan, 2008; Jeffrey et al., 2014). Whether with small or large student groups, most teachers in this study kept the flow throughout the semester, although with different kind of activities.

### Selection of teaching and learning activities

During synchronous meetings, promoting active learning either individually (e.g., problem solving) or collaboratively (e.g., debate) enhanced student behavioral and cognitive engagement, in line with findings of other publications in BL (e.g., Manwaring et al., 2017). Depending on whether the synchronous meetings happened face-to-face or online, on the group size, and sometimes on the course content, the kind of activities also varied. For instance, instructors in traditional BL courses often described role-playing games or simulations, while instructors in blended online or blended synchronous courses mostly relied on discussion activities with the students. Whether this could be explained by a lack of confidence or knowledge about digital opportunities relative to synchronous online activities is unknown. Mainly, teachers selected activities they were comfortable with, depending on their course content and group size. Even in blended synchronous courses where the group sizes were often very large, most teachers managed to keep students in action, for instance by alternating discussion in small and large groups or by using poll applications.

In asynchronous mode, strategies like using supportive digital tools and proposing a longitudinal assessment with several deliverables were emphasized to foster student behavioral and cognitive engagement. To our knowledge, it was the first time that the strategy consisting in engaging students in a longitudinal project throughout the semester was explicitly linked to student engagement in the BL literature, although it was repeatedly mentioned by instructors in this study. On another note, in undergraduate BL courses, our findings indicated that student behavioral engagement was specifically targeted by teachers through the use of digital tools and frequent online assessments. Enlarging the results of Vaughan (2014) relating to online collaborative learning applications in 1st-year BL courses, this study emphasized the use of various digital tools to promote student behavioral engagement in undergraduate BL courses, both synchronously and asynchronously, suggesting that students would hardly stay engaged otherwise.

Regarding the importance of demonstrating the relevance of activities, topics or resources to the students, teachers mainly targeted student cognitive and emotional engagement in graduate BL courses through experience-sharing and learning co-construction between students, in line with Taylor et al. (2019). They also enhanced the relevance of synchronous activities through integration, experience, and/or deepening of knowledge previously addressed asynchronously to foster student cognitive engagement, again recalling the importance of structuring the course such that synchronous and asynchronous activities complement each other (Garrison & Vaughan, 2008). Finally, providing choices to the students was mentioned by teachers to foster student emotional and cognitive engagement, echoing other publications in BL or other environments (e.g., Bolliger & Martin, 2018; Cundell & Sheepy, 2018; Montgomery et al., 2015). However, most teachers only provided choices in discussion or assignment topics, which they felt were sufficient to enhance students’ sense of control over activities and, consequently, their emotional and cognitive engagement.

While some findings relating to the selection of activities echoed known strategies (e.g., promoting active learning or offering choices) to foster student engagement in BL or other environments (e.g., Bolliger & Martin, 2018; Exeter et al., 2010; Manwaring et al., 2017; Taylor et al., 2019), this study provided further strategies and explicitly linked these to student engagement dimensions. This second meta-category also emphasized the need of carefully selecting activities in both asynchronous and synchronous modes of BL in order to maintain student engagement continuously throughout the semester, while illustrating the use of corresponding strategies in varied BL courses.

### Teacher’s role and course relationships

Here, the course beginning appeared of particular importance and was often initiated with a first synchronous (face-to-face or online) meeting. On the one hand, clearly communicating how the BL course would unfold, with both synchronous and asynchronous activities, was emphasized by teachers to promote student behavioral and emotional engagement, recalling the design and organization category of teaching presence in the Community of Inquiry Framework (Vaughan et al., 2013). Particularly, explanations about the purpose of asynchronous activities as well as corresponding expectations are needed to enhance student engagement in BL and so that they fully benefit from both modes. This realization echoed Shea et al. (2006), in suggesting that students may feel uncertain of what to do asynchronously online, which is why a clear communication of objectives and expectations is particularly important. On the other hand, teachers stressed out the need to create trusting relationships in the BL course from the beginning of the semester to enhance student emotional and cognitive engagement, for instance with ice-breaking or practice-sharing activities. Thereafter, personalized contacts with the teacher and peer-to-peer sharing activities supported their emotional and cognitive engagement. The previous findings were in line with other studies in BL or online learning (e.g., Berry, 2019; Bolliger & Martin, 2018; Lervik et al., 2018; Orcutt & Dringus, 2017; Robinson et al., 2017), and recalled the intersection of teaching and social presence of the Community of Inquiry Framework concerned with establishing a positive affective climate (Vaughan et al., 2013). In this regard, the study also highlighted some specificities regarding teachers’ strategies in undergraduate or graduate BL courses. At the undergraduate level, teachers spoke of taking a great deal of time to reassure students in order to foster their emotional and behavioral engagement. In contrast, teachers in graduate BL courses promoted student emotional and cognitive engagement by inviting them to find their own individual course goal at the beginning of the semester.

This third meta-category also highlighted the essential guiding role of the teacher to foster student engagement in BL courses, as also noted in other environments (e.g., Baldwin, 2019; Ma et al., 2015; Zepke et al., 2014). Recalling the facilitation strategies proposed by Martin et al. (2018) in online courses, the findings of this study further detailed the teacher’s guiding role in BL (e.g., recalling deadlines and what to focus on, realigning or relaunching discussions) and stressed its importance in both modes to foster student behavioral and emotional engagement. In undergraduate BL courses, especially, teachers emphasized that engaging students meant guiding and reassuring them step-by-step. Furthermore, reminding students of their presence and availability, particularly in asynchronous mode, fostered student emotional and cognitive engagement. These findings were in line with the study of Shea et al. (2006) showing that teachers’ active presence in asynchronous online courses promoted students’ sense of connectedness and learning. The “reassuring and supporting role” of the teacher in blended online courses was also emphasized in Farrell and Brunton's (2020, p. 9) study, in which students explained that teachers supported them by providing content clarification, encouragement, and guidance as regards learning strategies, thereby fostering their cognitive and emotional engagement. This study particularly emphasized that teachers showing students their presence and availability to support them, both synchronously and asynchronously, encouraged students to be there too and engage themselves in a BL course.

## Conclusion

In a recent review concerning BL, Graham (2019) stated that “much of the BL engagement research stays at a general level, not specifying pedagogical features that might impact engagement” (p. 16) and that a distinction should be made between teachers’ strategies in asynchronous and synchronous modes. Indeed, publications that specifically address student engagement in BL are rare, as are examinations of teachers’ strategies in such environments (Halverson & Graham, 2019; Smith & Hill, 2019; Taylor et al., 2018). This qualitative study answered these needs by investigating how teachers fostered student engagement in BL environments, i.e., blended, blended online, and blended synchronous courses. Thanks to a large data collection conducted in various disciplines, at the undergraduate and graduate levels, in four higher education institutions, the study proposed a broad and comprehensive examination of teachers’ strategies in this regard. Strategies to foster student engagement in BL courses were detailed and related to asynchronous or synchronous modes where appropriate. When possible, they were also linked to student behavioral, emotional, and cognitive engagement.

Overall, the findings of this study emphasized the importance of fully exploiting and integrating both modes in BL in order to optimize student engagement. Strategies were classified into three meta-categories and eight categories, as well as concretely illustrated in varied contexts, to guide practitioners and researchers toward enhanced student engagement in BL environments, whether asynchronously or synchronously. This study also provided suggestions for teachers relative to the findings of Rasheed et al. (2020) concerning teachers struggling with the online mode of traditional BL courses, wondering how to structure their courses, to guide students, and to increase the sense of closeness between students and teachers asynchronously online. While these findings provided courses of action for practitioners, they also yielded interesting avenues for future research. For instance, some specific traits shared at the undergraduate and graduate levels were highlighted. Given that studies on graduate BL courses are rare (Taylor et al., 2019), future studies could focus on student engagement in BL at the graduate level or contrast student engagement in BL at the graduate versus the undergraduate level. Future studies could also use the classification of strategies presented herein as a starting point to investigate student engagement in specific BL environments such as blended online or blended synchronous courses, concerning which little is known.

Finally, this study has some limitations. First of all, it did not present teachers’ discipline specific strategies, instead aiming at offering a broad picture of how teachers fostered student engagement in various disciplines. Future studies could explore how the teachers’ strategies herein described would be applied in selected disciplines in BL, or even contrast these strategies between disciplines. On another note, the fact that participants agreed to a single 1-h interview somewhat restricted the conversations. While the interviewer tried to obtain as rich information as possible, a second interview with the same participants would have allowed to deepen their explanations. Therefore, future research could explore more longitudinal designs involving repeated interviews with participants. Finally, the study investigated teachers’ strategies to foster student engagement rather than students’ perspectives in this regard. Indeed, it was designed to draw a descriptive portrait of the strategies used by teachers in blended courses, as the first stage of a doctoral research. In a subsequent stage of the doctoral research, the effects of categories of teachers’ strategies on student engagement dimensions were empirically investigated, according to the students’ themselves (Heilporn et al., 2021).

## Data Availability

The datasets used and/or analyzed during the current study are available from the corresponding author on reasonable request.

## Appendix A

Here are detailed course information for participants P1 to P20 (see Table 2).

**Table 2:** Detailed information on participants’ courses

	University level	BL environment	Discipline	Number of students*
P1	Graduate	TBL	Research methods in accounting	20
P2	Undergraduate	BSL	Marketing	60
P3	Graduate	BOL	Education	15
P4	Undergraduate	BSL	Sociology	50–80
P5	Undergraduate	BSL	Engineering Ethics	150–200
P6	Undergraduate	TBL	Management	45–50
P7	Graduate	BOL	Economy	7
P8	Undergraduate	TBL	Sustainable development in engineering	60–70
P9	Undergraduate	BOL	Sustainable development	40
P10	Undergraduate	TBL	Computer science	130
P11	Undergraduate	TBL	Management	45–65
P12	Undergraduate	TBL	Communication	50
P13	Undergraduate	BSL	Industrial relations	250
P14	Undergraduate	BOL	Mathematics	100–150
P15	Graduate	TBL	Management	30
P16	Undergraduate	BSL	Economy	300
P17	Graduate	TBL	Accounting	50
P18	Undergraduate	BSL	Information systems	50
P19	Graduate	TBL	Educational management	15–20
P20	Graduate	TBL	Education	25

*TBL* traditional blended learning, *BOL* blended online learning, *BSL* blended synchronous learning

*Approximatively per group

## Appendix B

The final coding scheme is presented below (see Tables 3, 4 and 5).

**Table 3:** Meta-category: course structure and pace

Main category and related strategies	Description
**Course_Structure**	**Teachers presenting a clear, continuous, and unified course structure**
Indications_WhenWhatWhere	Clear indications about activities (when/what/where)
Connections_SyncAsync	Explicit connections unifying asynchronous and synchronous modes
Continuity_ThroughoutSemester	Continuity between activities throughout the semester
**Course_Pace**	**Teachers ensuring a sustained pace throughout the course (synchronously and asynchronously)**
Async_DiversificationActivities	Diversity of asynchronous activities
Sync_NoDowntimeHighInteractions	Busy, dynamic synchronous meetings
*SyncOL_ShortDynamicMeetings	For BOL, BSL, short and very dynamic meetings

Boldface: meta-categories and categories of teachers’ strategies

Regular text: teachers’ strategies inside a category

*Sign: a strategy for a specific blended modality or at a specific university level

**Table 4:** Meta-category: selection of teaching and learning activities

Main category and related strategies	Description
**Activities_ActiveInteractive**	**Teachers proposing active or interactive teaching and learning activities**
Async_DigitalToolsSupport	Asynchronous use of digital tools
Sync_ActiveLearning	Synchronous active or interactive activities
LongitudinalAssessment	Use of a longitudinal assessment/project in several deliverables throughout the semester
*Undergrad_FrequentOLAssess	In undergraduate courses, frequent online assessments
*Undergrad_PollInteractiveApps	In undergraduate courses, use of poll applications (synchronously)
**ActivitiesTopicsResources_Relevance**	**Teachers selecting activities, topics, and resources that are relevant for students**
LinksWithPractice_ConcreteEx	Links with practice (or life) and concrete examples, expert participation
HotTopics_For_Debates	Selection of hot topics for stimulating debates
Sync_Deepens_Async	Enhanced relevance when synchronous meetings deepen previous asynchronous activities (apply, integrate, etc.)
*Grad_PersonalLearningGoal	In graduate courses, inviting students to find their own individual goal
*Grad_ExperienceSharing	In graduate courses, students share about their practice/experience
**TopicsOrOther_Choices**	**Teachers providing choices in topics, resources, or assignment formats**
ChoicesTopics	Students can choose their preferred topic for a discussion or assignment
ChoicesResources	Students have some choices in pedagogical resources
ChoicesFormats	Students can choose their preferred format for an assignment

Boldface: meta-categories and categories of teachers’ strategies

Regular text: teachers’ strategies inside a category

*Sign: a strategy for a specific blended modality or at a specific university level

**Table 5:** Meta-category: teacher’s role and course relationships

Main category and related strategies	Description
**Explicitation_Beginning**	**Teachers explaining course organizational aspects, objectives, and expectations (asynchronous and synchronous modes) at the beginning of the semester**
Explicitation_CourseOrganization	Teachers clearly explain organizational aspects for synchronous and asynchronous modes of blended courses
Explicitation_ExpectationsObjectives	Teachers clearly states course objectives and expectations for synchronous and asynchronous modes
**Relationships_TrustFromBeginning**	**Teachers establishing trusting relationships between students and with the teacher from the beginning of the semester**
InitiateRelation_SharingActivities	Use of ice-breaking or sharing activities at the beginning of the semester
PersonalizedContacts	Teacher has personalized contacts with students (use their names, discussions)
PeerSharingDuringSemester	Peer evaluation or other peer-to-peer sharing to enhance relations
**Guide_AsyncSync**	**Teachers guiding students throughout the semester, both synchronously and asynchronously**
ExplainGoals	Teachers clearly explain goals of activities
RecallDeadlinesOrOther	Teachers recall deadlines and what to focus on (often asynchronously)
QuestionRealignRelaunch	Teachers relaunch students during activities (questioning, realigning, challenging)
GlobalFeedback	Teachers provide feedback to the whole group of students
IndividualFeedback	Teachers provide individual feedback (text, audio, video)
RecallPresenceAvailability	Teachers explicitly recall their availability and presence to support students
IndividualSupportOnDemand	Teachers provide additional support to students when needed (outside regular contacts)

Boldface: meta-categories and categories of teachers’ strategies

Regular text: teachers’ strategies inside a category

*Sign: a strategy for a specific blended modality or at a specific university level

## References

[CR700] Allen, I. E. & Seaman, J. (2016). Online report card: Tracking online education in the United States. Babson Survey Research Group.

[CR2] Baldwin SJ (2019). Assimilation in online course design. American Journal of Distance Education.

[CR3] Bates T (2018). The 2017 national survey of online learning in Canadian post-secondary education: Methodology and results. International Journal of Educational Technology in Higher Education.

[CR4] Beatty, B. (2007). Transitioning to an online world: Using HyFlex courses to bridge the gap. *EdMedia: World Conference on Educational Media and Technology*, 2701–2706.

[CR5] Beatty, B. (2014). Hybrid courses with flexible participation: The HyFlex Course Design. In *Practical applications and experiences in K-20 blended learning environments* (Kyei-Blankson, L., pp. 153–177). IGI Global. 10.4018/978-1-4666-4912-5.ch011

[CR6] Beatty, B. J. (2019). *Hybrid-flexible course design*. EdTech Books. https://edtechbooks.org/hyflex/impact

[CR7] Berry, S. (2019). Teaching to connect: Community-building strategies for the virtual classroom. *Online Learning*, *23*(1), 164–183. 10.24059/olj.v23i1.1425

[CR8] Binnewies, S., & Wang, Z. (2019). Challenges of student equity and engagement in a HyFlex Course. In C. N. Allan, C. Campbell, & J. Crough (Eds.), *Blended learning designs in STEM higher education: Putting learning first* (pp. 209–230). Springer Singapore. 10.1007/978-981-13-6982-7_12

[CR9] Boelens, R., De Wever, B., & Voet, M. (2017). Four key challenges to the design of blended learning: A systematic literature review. *Educational Research Review*, *22*(Supplement C), 1–18. 10.1016/j.edurev.2017.06.001

[CR10] Boelens R, Voet M, De Wever B (2018). The design of blended learning in response to student diversity in higher education: Instructors’ views and use of differentiated instruction in blended learning. Computers & Education.

[CR11] Bolliger DU, Martin F (2018). Instructor and student perceptions of online student engagement strategies. Distance Education.

[CR12] Bond M, Bedenlier S (2019). Facilitating student engagement through educational technology: Towards a conceptual framework. Journal of Interactive Media in Education.

[CR13] Bond M, Buntins K, Bedenlier S, Zawacki-Richter O, Kerres M (2020). Mapping research in student engagement and educational technology in higher education: A systematic evidence map. International Journal of Educational Technology in Higher Education.

[CR600] Bonk, C. J., & Graham, C. R. (2012). *The handbook of blended learning: Global perspectives, local designs*. John Wiley & Sons.

[CR14] Borup J, Graham CR, West RE, Archambault L, Spring KJ (2020). Academic communities of engagement: An expansive lens for examining support structures in blended and online learning. Educational Technology Research and Development.

[CR15] Bower M, Dalgarno B, Kennedy GE, Lee MJW, Kenney J (2015). Design and implementation factors in blended synchronous learning environments: Outcomes from a cross-case analysis. Computers & Education.

[CR16] Bruggeman B, Tondeur J, Struyven K, Pynoo B, Garone A, Vanslambrouck S (2021). Experts speaking: Crucial teacher attributes for implementing blended learning in higher education. The Internet and Higher Education.

[CR17] Burke A (2019). Student retention models in higher education: A literature review. College and University.

[CR18] Christenson, S. L., Reschly, A. L., & Wylie, C. (Eds.). (2012). *Handbook of research on student engagement*. Springer US. 10.1007/978-1-4614-2018-7

[CR19] Cundell, A., & Sheepy, E. (2018). Student perceptions of the most effective and engaging online learning activities in a blended graduate seminar. *Online Learning*, *22*(3), 87–102. 10.24059/olj.v22i3.1467

[CR20] Drysdale JS, Graham CR, Spring KJ, Halverson LR (2013). An analysis of research trends in dissertations and theses studying blended learning. The Internet and Higher Education.

[CR21] Exeter DJ, Ameratunga S, Ratima M, Morton S, Dickson M, Hsu D, Jackson R (2010). Student engagement in very large classes: The teachers’ perspective. Studies in Higher Education.

[CR22] Farrell O, Brunton J (2020). A balancing act: A window into online student engagement experiences. International Journal of Educational Technology in Higher Education.

[CR23] Foogooa, R., & Ferdinand-James, D. (2017). Use of Facebook for enhancing student engagement in a higher education blended engineering course. *Innovative Issues and Approaches in Social Sciences*, *10*(1), 8–31. 10.12959/issn.1855-0541.IIASS-2017-no1-art1

[CR24] Fredricks JA, Blumenfeld PC, Paris AH (2004). School engagement: Potential of the concept, state of the evidence. Review of Educational Research.

[CR25] Fredricks JA, Filsecker M, Lawson MA (2016). Student engagement, context, and adjustment: Addressing definitional, measurement, and methodological issues. Learning and Instruction.

[CR26] Fredricks, J. A., Reschly, A. L., & Christenson, S. L. (2019). Interventions for student engagement: Overview and state of the field. In *Handbook of student engagement interventions* (pp. 1–11). Elsevier. 10.1016/B978-0-12-813413-9.00001-2

[CR27] Garrison DR, Vaughan ND (2008). Blended learning in higher education: Framework, principles, and guidelines.

[CR28] Graham, C. R. (2019). Current research in blended learning. In M. G. Moore, & W. C. Diehl (Eds.), *Handbook of distance education* (4th ed., pp. 173–188). Routledge. 10.4324/9781315296135-15

[CR29] Graham, C. R., Henrie, C. R., & Gibbons, A. S. (2014). Developing models and theory for blended learning research. In A. G. Picciano, C. D. Dziuban, & C. R. Graham (Eds.), *Blended learning: Research perspectives* (Vol. 2, pp. 13–33). Routledge.

[CR30] Halverson, L. R., & Graham, C. R. (2019). Learner engagement in blended learning environments: A conceptual framework. *Online Learning*, *23*(2), 145–178. 10.24059/olj.v23i2.1481

[CR31] Halverson LR, Graham CR, Spring KJ, Drysdale JS, Henrie CR (2014). A thematic analysis of the most highly cited scholarship in the first decade of blended learning research. The Internet and Higher Education.

[CR32] Heilporn, G., & Lakhal, S. (2020). Fostering student engagement in blended courses: A qualitative study at the graduate level in a business faculty [Manuscript submitted for publication]. Faculty of Education, Université de Sherbrooke.

[CR33] Heilporn, G., Lakhal, S., & Bélisle, M. (2021). Relationships, relevance and sustained pace are key to foster student engagement in blended online courses [Manuscript submitted for publication]. Faculty of Education, Université de Sherbrooke.

[CR34] Henrie CR, Bodily R, Manwaring KC, Graham CR (2015). Exploring intensive longitudinal measures of student engagement in blended learning. International Review of Research in Open & Distance Learning.

[CR35] Jeffrey LM, Milne J, Suddaby G, Higgins A (2014). Blended learning: How teachers balance the blend of online and classroom components. Journal of Information Technology Education.

[CR36] Johnson, N. (2019). *National survey of online and digital learning 2019 national report*. Canadian Digital Learning Research Association.

[CR37] Kahu ER (2013). Framing student engagement in higher education. Studies in Higher Education.

[CR38] Kahu ER, Nelson K (2018). Student engagement in the educational interface: Understanding the mechanisms of student success. Higher Education Research & Development.

[CR39] Kulkarni AK, Iwinski T (2016). Enhancing student engagement in a blended resident and online course using clickers and embedded questions. Journal of Engineering Education Transformations.

[CR40] Lakhal S, Bateman D, Bédard J (2017). Blended synchronous delivery mode in graduate programs: A literature review and its implementation in the master teacher program. Collected Essays on Learning and Teaching.

[CR41] Lakhal S, Bélisle M (2020). A continuum of blended and online learning. The Canadian Journal for the Scholarship of Teaching and Learning.

[CR42] Lakhal, S., & Meyer, F. (2019). Blended learning. In A. Tatnall (Ed.), *Encyclopedia of education and information technologies*. Springer.

[CR43] Lakhal S, Mukamurera J, Bédard M-E, Heilporn G, Chauret M (2020). Features fostering academic and social integration in blended synchronous courses in graduate programs. International Journal of Educational Technology in Higher Education.

[CR44] Lawson MA, Lawson HA (2013). New conceptual frameworks for student engagement research, policy, and practice. Review of Educational Research.

[CR45] Lee J-S (2014). The relationship between student engagement and academic performance: Is it a myth or reality?. The Journal of Educational Research.

[CR46] Lervik MJ, Vold T, Holen S (2018). Conditions for cooperating and dialogue through the utilization of technology in online education. Universal Journal of Educational Research.

[CR47] Ma J, Han X, Yang J, Cheng J (2015). Examining the necessary condition for engagement in an online learning environment based on learning analytics approach: The role of the instructor. The Internet and Higher Education.

[CR48] Mandernach BJ (2015). Assessment of student engagement in higher education: A synthesis of literature and assessment tools. International Journal of Learning, Teaching and Educational Research.

[CR49] Manwaring, K. C., Larsen, R., Graham, C. R., Henrie, C. R., & Halverson, L. R. (2017). Investigating student engagement in blended learning settings using experience sampling and structural equation modeling. *The Internet and Higher Education*, *35*(Supplement C), 21–33. 10.1016/j.iheduc.2017.06.002

[CR50] Martin F, Ahlgrim-Delzell L, Budhrani K (2017). Systematic review of two decades (1995 to 2014) of research on synchronous online learning. American Journal of Distance Education.

[CR51] Martin F, Wang C, Sadaf A (2018). Student perception of helpfulness of facilitation strategies that enhance instructor presence, connectedness, engagement and learning in online courses. The Internet and Higher Education.

[CR52] McGee P, Reis A (2012). Blended course design: A synthesis of best practices. Journal of Asynchronous Learning Networks.

[CR53] Merriam, S. B., & Tisdell, E. J. (2015). *Qualitative research: A guide to design and implementation*. Wiley.

[CR54] Miles MB, Huberman AM, Saldaña J (2020). Qualitative data analysis: A methods sourcebook (4th ed.).

[CR55] Montgomery AP, Hayward DV, Dunn W, Carbonaro M, Amrhein CG (2015). Blending for student engagement: Lessons learned for MOOCs and beyond. Australasian Journal of Educational Technology.

[CR56] Orcutt JM, Dringus LP (2017). Beyond being there: Practices that establish presence, engage students and influence intellectual curiosity in a structured online learning environment. Online Learning.

[CR57] Picciano, A. G. (2009). Blending with purpose: The multimodal model. *Journal of Asynchronous Learning Networks*, *13*(1), 7–18. 10.24059/olj.v13i1.1673

[CR58] Pima JM, Odetayo M, Iqbal R, Sedoyeka E (2018). A thematic review of blended learning in higher education. International Journal of Mobile and Blended Learning (IJMBL).

[CR59] Power M (2008). The emergence of a blended online learning environment. MERLOT Journal of Online Learning and Teaching.

[CR60] Power M, Vaughan N (2010). Redesigning online learning for international graduate seminar delivery. Journal of Distance Education.

[CR61] Raes A, Detienne L, Windey I, Depaepe F (2019). A systematic literature review on synchronous hybrid learning: Gaps identified. Learning Environments Research.

[CR62] Raes A, Vanneste P, Pieters M, Windey I, Van Den Noortgate W, Depaepe F (2020). Learning and instruction in the hybrid virtual classroom: An investigation of students’ engagement and the effect of quizzes. Computers & Education.

[CR63] Rasheed RA, Kamsin A, Abdullah NA (2020). Challenges in the online component of blended learning: A systematic review. Computers & Education.

[CR64] Reschly, A. L., & Christenson, S. L. (2012). Jingle, jangle, and conceptual haziness: Evolution and future directions of the engagement construct. In S. L. Christenson, A. L. Reschly, & C. Wylie (Eds.), *Handbook of research on student engagement* (pp. 3–19). Springer. 10.1007/978-1-4614-2018-7_1

[CR65] Robinson, H. A., Kilgore, W., & Warren, S. J. (2017). Care, communication, support: Core for designing meaningful online collaborative learning. *Online Learning*, *21*(4), 29–51. 10.24059/olj.v21i4.1240

[CR66] Schindler LA, Burkholder GJ, Morad OA, Marsh C (2017). Computer-based technology and student engagement: A critical review of the literature. International Journal of Educational Technology in Higher Education.

[CR67] Seaman, J. E., Allen, I. E., & Seaman, J. (2018). *Grade increase: Tracking distance education in the United States*. Babson Survey Research Group.

[CR68] Serrano DR, Dea-Ayuela MA, Gonzalez-Burgos E, Serrano-Gil A, Lalatsa K (2019). Technology enhanced learning in higher education: How to enhance student engagement through blended learning. European Journal of Education.

[CR69] Shea P, Sau Li C, Pickett A (2006). A study of teaching presence and student sense of learning community in fully online and web-enhanced college courses. The Internet and Higher Education.

[CR70] Siemens, G., Gašević, D., & Dawson, S. (2015). *Preparing for the digital university: A review of the history and current state of distance, blended, and online learning*. http://linkresearchlab.org/PreparingDigitalUniversity.pdf

[CR71] Sinatra GM, Heddy BC, Lombardi D (2015). The challenges of defining and measuring student engagement in science. Educational Psychologist.

[CR72] Skinner, E. A., & Pitzer, J. R. (2012). Developmental dynamics of student engagement, coping, and everyday resilience. In S. L. Christenson, A. L. Reschly, & C. Wylie (Eds.), *Handbook of research on student engagement* (pp. 21–44). Springer US. 10.1007/978-1-4614-2018-7_2

[CR73] Smith K, Hill J (2019). Defining the nature of blended learning through its depiction in current research. Higher Education Research & Development.

[CR74] Spring KJ, Graham CR, Hadlock CA (2016). The current landscape of international blended learning. International Journal of Technology Enhanced Learning.

[CR75] Tan, M., & Hew, K. F. (2016). Incorporating meaningful gamification in a blended learning research methods class: Examining student learning, engagement, and affective outcomes. *Australasian Journal of Educational Technology*, *32*(5), 19–34. 10.14742/ajet.2232

[CR76] Taylor MC, Atas S, Ghani S (2019). Alternate dimensions of cognitive presence for blended learning in higher education. International Journal of Mobile and Blended Learning (IJMBL).

[CR77] Taylor M, Vaughan N, Ghani SK, Atas S, Fairbrother M (2018). Looking back and looking forward: A glimpse of blended learning in higher education from 2007–2017. International Journal of Adult Vocational Education and Technology (IJAVET).

[CR78] Thomas DR (2006). A general inductive approach for analyzing qualitative evaluation data. American Journal of Evaluation.

[CR79] Torrisi-Steele G, Drew S (2013). The literature landscape of blended learning in higher education: The need for better understanding of academic blended practice. International Journal for Academic Development.

[CR500] Truhlar AM, Williams KM, Walter MT (2018). Case study: Student engagement with course content and peers in synchronous online discussions. Online Learning.

[CR501] Vaughan N (2014). Student engagement and blended learning: Making the assessment connection. Education Sciences.

[CR80] Vaughan, N., Cleveland-Innes, M., & Garrison, R. (2013). *Teaching in blended learning environments: Creating and sustaining communities of inquiry*. Athabasca University Press. http://www.aupress.ca/index.php/books/120229.

[CR81] Zepke N, Leach L, Butler P (2014). Student engagement: Students’ and teachers’ perceptions. Higher Education Research & Development.

